# Editorial: Data-driven clinical biosignatures and treatment for neurodegenerative diseases

**DOI:** 10.3389/fnins.2023.1171788

**Published:** 2023-03-28

**Authors:** Nizhuan Wang, Lei Chen, Wei Kong, Chung Y. Hsu, I-Shiang Tzeng

**Affiliations:** ^1^School of Biomedical Engineering, ShanghaiTech University, Shanghai, China; ^2^College of Information Engineering, Shanghai Maritime University, Shanghai, China; ^3^Graduate Institute of Clinical Medical Science, College of Medicine, China Medical University, Taichung, Taiwan; ^4^Department of Research, Cooperative Research Center, Taipei Tzu Chi Hospital, Buddhist Tzu Chi Medical Foundation, New Taipei City, Taiwan

**Keywords:** data-driven, biosignature, neurodegenerative diseases, brain structure, treatment, association rule analysis

Illnesses known as neurodegenerative diseases, such as Alzheimer's disease and Parkinson's disease, result in the death of specific brain tissues (Gammon, 2014). Biosignatures are associated with neurodegenerative diseases (Li et al., 2021). To date, neurodegenerative diseases have been diagnosed and treated according to reliable biosignatures (Karaboğa and Sezgintürk, 2022), including those obtained from neuropsychological testing (Lin and McDonough, 2022) and serological testing, such as magnetic resonance imaging findings, blood counts, and thyroid-stimulating hormone, vitamin B-12, and folic acid levels. In addition, amyloid beta and phosphorylated tau are molecular trait data that are obtained through data-driven computing techniques with the aim of understanding the mechanisms of brain diseases (Zhang et al., 2021) and overcoming challenges in clinical practice.

Thus, there is an urgent need to investigate trustworthy biosignatures for diagnosis and treatment through advances in data-driven computation involving dry and wet laboratory methods. We considered the present Research Topic on “*Data-driven clinical biosignatures and treatment for neurodegenerative diseases*” by Frontiers in Neuroscience, and we have provided here updates and unique viewpoints on this urgent subject from the findings of seven papers. Among these papers, one is a trial study, one is a prospective study, two are retrospective cohort studies, two are secondary analysis studies based on acupoints, and one is a study of machine learning algorithm application under several brain structure datasets. Overall, the studies focus on exploring reliable biosignatures, such as novel DNA methylation loci, and beneficial acupoints for the treatment of neurodegenerative diseases from data-driven perspectives. In addition, research on Baduanjin exercise, which has a positive impact on non-motor symptoms, has been elucidated.

In the first study, Dong et al. conducted a single-center and self-controlled trial in China to investigate whether Parkinson's disease patients benefited from Baduanjin exercise, especially in relation to their non-motor symptoms, balance, gait, and everyday activities. Significant evidence was found to support the improvement of several indices after Baduanjin exercise. Therefore, Baduanjin exercise is recommended as a simple, inexpensive, and efficient treatment to alleviate the symptoms of Parkinson's disease patients. At present, early treatment initiation for neurological function recovery is also urgent for stroke patients. Developing alternative interventions combined with rehabilitation management may improve the quality of daily life or even postpone medication treatment for these patients. Tseng et al. reviewed cohort data retrospectively from the electronic medical records of Taipei Tzu Chi Hospital to explore the effects of acupuncture and traditional Chinese herbal medicine combined with clinical rehabilitation on the Barthel Index scores and National Institutes of Health Stroke Scale. After such intervention in the early subacute phase, the functional recovery of the stroke patients showed significant improvement. In another retrospective cohort study, Lu et al. examined the effects of local anesthetic and general anesthesia operational strategies for clinical symptoms alleviation in patients with Parkinson's disease, such as motor and non-motor symptoms, after subthalamic nucleus deep brain stimulation surgery at the Affiliated Brain Hospital of Nanjing Medical University. In terms of the surgical time and intracranial volume, as well as the intraoperative microelectrode recording signal length, general anesthesia operation methods appeared to be superior. Deep brain stimulation under general anesthesia will gain popularity, as electrode precision may be ensured by intraoperative microelectrode recording and signal monitoring. Recently, data mining approaches applied for acupoint secondary data analysis have become popular in traditional Chinese medicine. A two-paper series by Wang et al. and Wang et al. addressed this by reporting on the results of numerous randomized controlled trials on the impact of kernel combinations of acupoints on Alzheimer's disease and stroke patients. *A priori* algorithm-based association rule analysis is suitable for examining the underlying principles of acupuncture point/location selections. The greatest impact of kernel combinations of acupoints was investigated with the criteria of contained support, confidence, and lift. The results of both papers recommend a core combination of acupoints for patients based on advanced data-driven technology. Another study also adopted a machine learning algorithm application based on several brain structure datasets. DNA methylation is a kind of biosignature for which the most effective regulatory roles are played during Alzheimer's disease pathogenesis. The assumed hypothesis was that the various brain structures may exhibit different pathological methylation changes in Alzheimer's disease patients. Li et al. adopted three classification algorithms, including partial decision tree, support vector machine, and random forest, to identify candidate Alzheimer's disease-associated methylation features with structural specificity. Several groups of quantitative rules were searched to demonstrate the consequences of DNA methylation in different brain areas (i.e., cerebellum, entorhinal cortex, dorsolateral prefrontal cortex, and hippocampus brain structures) on Alzheimer's disease pathogenesis. This research was the first to discover a subset of brain structure-specific Alzheimer's disease biosignatures and rules of quantification at the methylation level. Finally, a prospective study was conducted by Cai et al., because Parkinson's disease has unknown mechanisms that may affect the pedunculopontine nucleus and cause gait disability. Cai et al. prospectively recruited patients to investigate whether the motor performance and functional connection within the pedunculopontine nucleus changed after exercise. They found that the walking exercise altered the functional connection within the pedunculopontine nucleus in both a dose-independent (focused on the right pedunculopontine nucleus and the laterality of pedunculopontine nucleus connectivity strength) and dose-dependent (focused on the left pedunculopontine nucleus) manner. The pedunculopontine nucleus may participate in compensatory and pathological Parkinson's disease gait mechanisms.

## Author contributions

NW and I-ST wrote the draft. LC edited the language. WK, CH, and I-ST reviewed and revised the manuscript. All authors listed have made substantial, direct, and intellectual contributions to the work and approved it for publication.
